# Molecular evidence for the presence of malaria vector species a of the *Anopheles annularis *complex in Sri Lanka

**DOI:** 10.1186/1756-3305-4-239

**Published:** 2011-12-22

**Authors:** Sinnathamby N Surendran, Kanapathy Gajapathy, Vaitheki Kumaran, Tharmasegaram Tharmatha, Pavilupillai J Jude, Ranjan Ramasamy

**Affiliations:** 1 Department of Zoology, Faculty of Science, University of Jaffna, Jaffna 40000, Sri Lanka; 2 PAPSRB Institute of Health Sciences, Universiti Brunei Darussalam, Gadong BE 1410, Brunei Darussalam

**Keywords:** *Anopheles annularis*, malaria vector, ribosomal genes, sibling species, Southeast Asia, Sri Lanka

## Abstract

**Background:**

*Anopheles annularis s.l*. is a wide spread malaria vector in South and Southeast Asia, including Sri Lanka. The taxon *An. annularis *is a complex of two sibling species viz. A and B, that are differentiated by chromosome banding patterns and ribosomal gene sequences in India. Only species A is reported to be a malaria vector in India while the occurrence of sibling species in Sri Lanka has not been documented previously.

**Findings:**

Anopheline larvae were collected at a site in the Jaffna district, which lies within the dry zone of Sri Lanka, and reared in the laboratory. Emerged adults were identified using standard keys. DNA sequences of the D3 domain of 28S ribosomal DNA (rDNA) and the internal transcribed spacer-2 (ITS-2) of the morphologically identified *An. annularis *were determined. BLASTn searches against corresponding *An. annularis *sequences in GenBank and construction of phylogenetic trees from D3 and ITS-2 rDNA sequences showed that the Sri Lankan specimens, and *An. annularis s.l*. specimens from several Southeast Asian countries were closely related to species A of the Indian *An. annularis *complex.

**Conclusions:**

The results show the presence of the malaria vector *An. annularis *species A in Sri Lanka and Southeast Asia. Because *An. annularis *vectors have been long associated with malaria transmission in irrigated agricultural areas in the Sri Lankan dry zone, continued monitoring of *An. annularis *populations, and their sibling species status, in these areas need to be integral to malaria control and eradication efforts in the island.

## Findings

*Anopheles *(*Cellia*) *annularis *Van der Wulp is one of the species that form the "Annularis Group" that are classified under the Neocellia series of the subgenus *Cellia *[1]. The other members of this group are *An. nivipes, An. philippinensis, An. pallidus *and *An. schueffneri*. *An. annularis s.l*. is widespread in Asia from the Indian subcontinent to Southeast Asia, and has been incriminated as vector of malaria in India, Bangladesh, Myanmar, Malaysia and China [2-5]. The taxon *An. annularis *is reported to be a species complex comprising two sibling species *viz*. A and B in India [2,4]. The two sibling species can be distinguished through characteristic banding patterns in the polytene chromosome arm 2 [2]. They also possess different sequences in the D3 region of 28S RNA and the ITS-2 internally transcribed spacer region of ribosomal RNA [3]

*An. annularis s.l*. was previously reported to be an efficient local vector in a large irrigation project in the North-Central province in the dry zone of Sri Lanka [6]. In India species A is incriminated as a malaria vector while species B is a non-vector [4]. The Jaffna district, located in the dry zone of Sri Lanka, is an agricultural area that has been traditionally endemic for malaria but has seen the incidence decrease rapidly in the past few years due to the implementation of efficient malaria control measures [7]. An understanding of bionomics of different anopheline vectors in Jaffna and elsewhere in Sri Lanka is however needed for sustaining the control of malaria in the island. We therefore characterised the sibling species status of *An. annularis *specimens collected in the Jaffna district based on DNA sequences of ribosomal RNA genes.

**Figure 1:**
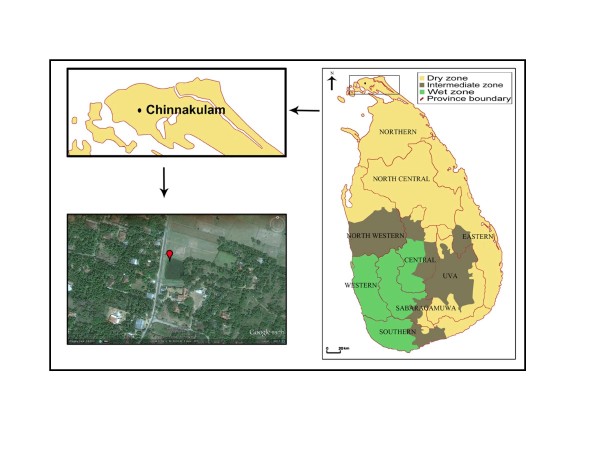
**Mosquito collection site in the district of Jaffna in the Northern province of Sri Lanka**. Larvae were collected from Chinnakkulam pond used to irrigate adjacent agricultural lands.

Anopheline larvae were collected using dippers as described earlier [8] during May and August 2011 from Chinnakulam pond (9°44^"^33.02^' ^N: 80°00^"^33.11^' ^E) in Chunnakam in the Jaffna district of the northern dry zone of Sri Lanka [Figure 1]. Collected larvae were brought to the Zoology Laboratory of the University of Jaffna and reared under laboratory conditions (28° ± 2°C) until they emerge as adults. Larvae were fed locally available powered fish meal pellets. Emerged adults were identified using standard keys [9,10]. Identified *An. annularis *and *An. pallidus *were preserved in isopropanol for subsequent molecular characterization.

The DNA from individual mosquitoes was extracted as described previously [11] with slight modification. Individual mosquitoes were homogenized with a plastic tissue grinder in 1.5 ml microcentrifuge tubes. 100 μl LIVAK extraction buffer (80 mM NaCl, 5.48% w/v sucrose, 1.57% w/v Tris-base, 50.8 mM EDTA, 05% w/v SDS) was added and samples were kept at 65°C for 30 min. Following the addition of 14 μl of 8 M potassium acetate, the homogenates were incubated on ice for 30 min and then centrifuged at 4°C for 20 min at 13, 000 rpm. Supernatants were recovered and DNA was precipitated overnight by the addition of 400 μl ethanol, then centrifuged at 4°C for 15 min at 13,000 rpm. The supernatant was discarded and DNA pellets were rinsed in 100 μl cold 70% ethanol, then centrifuged at 4°C for 5 min at 13, 000 rpm. Pellets were dried and resuspended in 50 μl TE buffer (pH 8.0). The ITS-2 region of the rDNA was amplified using primers ITS-2A (5^' ^- TGT GAA CTG CAG GAC ACA T- 3^'^) and ITS-2B (5^'^- TAT GCT TAA ATT CAG GGG GT - 3^'^) [12]. The D3 domain of 28S rDNA was amplified using D3A (5'-GAC CCG TCT TGA AAC ACG GA-3') and D3B (5^'^- TCG GAA GGA ACC AGT TAC TA - 3^'^) primers [13]. For each PCR assay, 50 μl PCR reaction mixture was used. Each mixture contained 0.50 μM of each primer, 200 μM of each dNTP, 1.5 mM of MgCl_2 _and 1.25 unit of Taq polymerase (Promega, USA). The PCR conditions for both PCRs (GeneAmp PCR System 9600, Applied Biosystems) were an initial denaturation at 95°C for 5 min followed by 35 cycles of 95°C for 30 s, 55°C for 30 s, and 72°C for 45 s followed by a final extension at 72°C for 7 min. The PCR products that were successfully amplified were then purified using the Quiaquik PCR Purification Kit (Qiagen, California, USA) to remove unincorporated primers and dNTPs prior to sequencing. The purified PCR amplicons were sent to M/s GENETECH, Sri Lanka for sequencing in both the forward and reverse directions. The derived sequences obtained were then analysed together with other representative sequences obtained from GenBank using ClustalW. Neighbour-Joining phylogeny tree was constructed with Bootstrap values from 500 replicates using the MEGA software [14].

Sequences of D3 and ITS-2 were derived from three *An. annularis *(annularis- Sri Lanka -1,2,3) and one *An. pallidus *(pallidus - Sri Lanka-1) specimens. These sequences were then compared by BLASTn searches against the corresponding Indian sequences in GenBank. The best match for D3 of the Sri Lankan *An. annularis *was with *An. annularis *species A of India with 100% identity over 352 bp (E-value 0) and for ITS-2 with *An. annularis *species A of India with 100% identity over 347 bp (E-value 0). The DNA sequences of specimens collected in this study in Jaffna have been deposited in GenBank with details as follows: D3 (JQ268278-annularis- Sri Lanka -1; JQ268279- annularis- Sri Lanka -3; JQ268281-pallidus- Sri Lanka -1) and ITS-2 (JQ268277-annularis- Sri Lanka -1; JQ268280 - annularis - Sri Lanka -3; JQ268282-pallidus- Sri Lanka -1).

**Figure 2:**
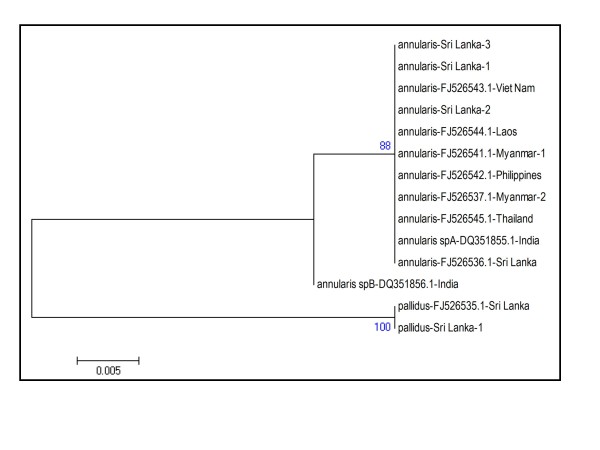
**Phylogenetic analysis based on D3 region of rDNA**. Sri Lanka - 1, 2 and 3 refers to individual specimens collected in this study. Other anopheline sequences from different countries were obtained from GenBank.

Construction of a phylogenetic tree based on D3 rDNA sequences resulted in two *An. annularis *clades [Figure 2]. One clade consisted D3 sequences from mosquitoes identified as sibling species A of the *An. annularis *in India, six *An. annularis s.l*. specimens from different Southeast Asian countries, one specimen of *An. annularis s.l*. collected previously in Sri Lanka (FJ526536.1) and the three *An. annularis *specimens from Jaffna. This clade is termed as the species A clade. The second related *An. annularis *clade contained a single sequence from sibling species B of *An. annularis *of India and is termed the species B clade. The differences between species A and B of India were caused by two substitutions [3]. The D3 sequences of Sri Lankan samples [present study and the voucher *An. annularis s.l*. specimen from Sri Lanka (FJ526536.1)] showed the same substitutions fixed for species A [Additional File 1].

An *An. pallidus *voucher specimen collected from Sri Lanka (FJ526535.1) and a specimen from the present study formed a distinct third clade in the D3 analysis [Figure 2].

**Figure 3:**
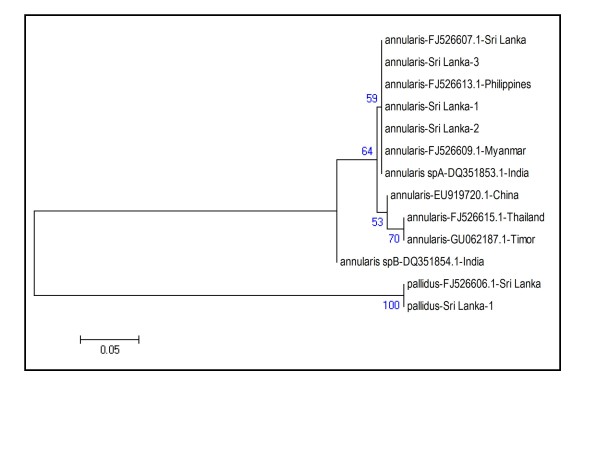
**Phylogenetic analysis based on ITS-2 region of rDNA**. Sri Lanka - 1, 2 and 3 refers to individual specimens collected in this study. Other anopheline sequences from different countries were obtained from GenBank.

Sequence analysis of the ITS-2 sequences of *An. annularis *collected during the present study and other available GenBank sequences for *An. annularis s.l*. were also carried out. The phylogenetic analysis showed that the three *An. annularis *from Jaffna formed a clade with *An. annularis *species A specimens from India and other *An. annularis s.l*. specimens from Southeast Asia and one from Sri Lanka [Figure 3]. This clade is again termed as the species A clade. A second related clade was represented by a single *An. annularis *species B specimen from India. The difference between species A and B of India was caused by seven substitutions and four indel events [3]. The reported substitutions and indels found in species A of India were present in the Sri Lankan samples [specimens of present study and a voucher specimen from Sri Lanka (FJ526607.1)] [Additional File 2].

The ITS-2 sequence from *An. pallidus *(FJJF526606.1) a voucher specimen from Sri Lanka and the *An. pallidus *specimen from Jaffna formed a third separate clade [Figure 3]. The sequence data from D3 and ITS-2 regions therefore confirm the morphological identification of *An. pallidus *in Sri Lanka.

Our findings show that *An. annularis *species A, confirmed as a vector species in India, is also present in the Jaffna district. The phylogenetic analysis further reveals that vector species A is widespread and can therefore play a role in malaria transmission in Southeast Asia.

A 1990 study at Weheragala, an agricultural settlement close to newly constructed irrigation canals of the Mahaweli river development project in the Sri Lankan dry zone, showed that *An. annularis s.l*. functioned as the predominant local vector, accounting for > 75% of the human-biting anopheline collection [6]. A very high entomological inoculation rate (EIR) for *Plasmodium vivax *of 0.12 infective bites per hour during the peak transmission season was reported for *An. annularis *at Weheragala [6]. The corresponding EIRs for *An. culicifacies*, the major vector of malaria elsewhere in Sri Lanka, were 0.06 and 0.04 for *Plasmodium vivax *and *P. falciparum *during the peak transmission season at Weheragala [6]. These high local malaria transmission rates, characterised by parasite prevalence rates of 20% in the population, may have initiated or contributed to the malaria epidemics in the country at that time. The present results are compatible with the suggestion that *An. annularis *species A may have been the relevant sibling species contributing to past malaria transmission in Weheragala and elsewhere in the Sri Lankan dry zone.

In some Indian states, e.g. Uttar Pradesh, *An. annularis *species A and B are sympatric with high zoophilic behavior and yet neither has yet been incriminated as vector. However in the states of Assam and Orissa only species A is present and implicated as a vector [2]. Both species are confined to riverine and canal-irrigated ecosystems in India. However presence of species A is also reported in hilly areas [2]. In this context, it will be useful to investigate the presence of *An. annularis *sibling species in the extensive hill country of central Sri Lanka.

The malaria incidence in Sri Lanka has fallen sharply from 210,039 cases in the year 2000 to 558 cases in 2009 [7]. The Ministry of Health plans to eliminate malaria from the country by 2015. However in the post-civil war era in Sri Lanka, there is resettlement of malaria-naïve persons and an expansion of agriculture in the dry zone. The presence of *An. annularis *species A in Sri Lanka, with its ability to undergo preimaginal development in the irrigated parts of the dry zone is therefore an important factor to be considered in planning for malaria eradication and control in the island.

The present study documents the presence of the malaria vector *An. annularis *species A in Sri Lanka and elsewhere in Southeast Asia. Because of the reported association of *An. annularis *vectors with malaria transmission in irrigated agricultural areas in the Sri Lankan dry zone, continued monitoring of *An. annularis *populations in these areas need to be integral to malaria control and eradication efforts in the island.

## Competing interests

The authors declare that they have no competing interests.

## Authors' contributions

SNS and RR conceived the study. TT and PJJ performed field collections. PJJ and SNS did mosquito identification. KG and VK did laboratory studies. SNS and RR did sequence analysis. SNS and RR wrote the manuscript. All authors read and approved the final manuscript.

## Supplementary Material

Additional file 1 **D3 sequences used for phylogenetic analysis**. Sequences of the D3 region of 28S rDNA used for phylogenetic analysis presented in Figure 1. Sri Lanka - 1, 2 and 3 refers to individual specimens collected in this study. Other sequences of *An. pallidus *and *An. annularis *from different countries were obtained from GenBank. Click here for file

Additional file 2 **ITS-2 sequences used for phylogenetic analysis**. ITS-2 sequences used for phylogenetic analysis presented in Figure 2. Sri Lanka - 1, 2 and 3 refers to individual specimens collected in this study. Other sequences of *An. pallidus *and *An. annularis *from different countries were obtained from GenBank. Click here for file
