# Supplementary material for: Molecular evidence for the presence of malaria vector species a of the Anopheles annularis complex in Sri Lanka
Source: Parasit Vectors. 2011 Dec 22;4:239. doi: 10.1186/1756-3305-4-239 (PMC3293028; doi:10.1186/1756-3305-4-239)
Supplement: Additional file 1 — D3 sequences used for phylogenetic analysis. Sequences of the D3 region of 28S rDNA used for phylogenetic analysis presented in Figure 1. Sri Lanka - 1, 2 and 3 refers to individual specimens collected in this study. Other sequences of An. pallidus and An. annularis from different countries were obtained from GenBank. [file 1756-3305-4-239-S1.DOC]

**Supplementary File 1: D3 sequences used for phylogenetic analysis**

annularis-FJ526544.1-Laos -------------------------------------GTA [ 40]

annularis-FJ526541.1-Myanmar-1 -------------------------------------... [ 40]

annularis-FJ526537.1-Myanmar-2 -------------------------------------... [ 40]

annularis-FJ526542.1-Philippines -------------------------------------... [ 40]

annularis-FJ526545.1-Thailand -------------------------------------... [ 40]

annularis-FJ526543.1-Viet_Nam -------------------------------------... [ 40]

annularis-FJ526536.1-Sri_Lanka -------------------------------------... [ 40]

annularis-Sri_Lanka-3 -------------------------------------... [ 40]

annularis-Sri_Lanka-1 -------------------------------------... [ 40]

annularis-Sri_Lanka-2 -------------------------------------... [ 40]

annularis_spA-DQ351855.1-India -------------------------------------... [ 40]

annularis_spB-DQ351856.1-India -------------------------------------..G [ 40]

pallidus-FJ526535.1-Sri_Lanka -------------------------------------..G [ 40]

pallidus-Sri_Lanka-1 ACGGTAGTATGTTCTTGCGCAAGCCAATGGGTGTCGC..G [ 40]

annularis-FJ526544.1-Laos GTACCGGCGTGTACTGCGCCCATATATAAACCCCACAGGC [ 80]

annularis-FJ526541.1-Myanmar-1 ........................................ [ 80]

annularis-FJ526537.1-Myanmar-2 ........................................ [ 80]

annularis-FJ526542.1-Philippines ........................................ [ 80]

annularis-FJ526545.1-Thailand ........................................ [ 80]

annularis-FJ526543.1-Viet_Nam ........................................ [ 80]

annularis-FJ526536.1-Sri_Lanka ........................................ [ 80]

annularis-Sri_Lanka-3 ........................................ [ 80]

annularis-Sri_Lanka-1 ........................................ [ 80]

annularis-Sri_Lanka-2 ........................................ [ 80]

annularis_spA-DQ351855.1-India ........................................ [ 80]

annularis_spB-DQ351856.1-India ........................................ [ 80]

pallidus-FJ526535.1-Sri_Lanka ..G.TA-....C.......A.................... [ 80]

pallidus-Sri_Lanka-1 ..G.TA-....C.......A.................... [ 80]

annularis-FJ526544.1-Laos GTAGACAACTCGAACAATGTCTGAGGGATTACGGGTTCGG [120]

annularis-FJ526541.1-Myanmar-1 ........................................ [120]

annularis-FJ526537.1-Myanmar-2 ........................................ [120]

annularis-FJ526542.1-Philippines ........................................ [120]

annularis-FJ526545.1-Thailand ........................................ [120]

annularis-FJ526543.1-Viet_Nam ........................................ [120]

annularis-FJ526536.1-Sri_Lanka ........................................ [120]

annularis-Sri_Lanka-3 ........................................ [120]

annularis-Sri_Lanka-1 ........................................ [120]

annularis-Sri_Lanka-2 ........................................ [120]

annularis_spA-DQ351855.1-India ........................................ [120]

annularis_spB-DQ351856.1-India ........................................ [120]

pallidus-FJ526535.1-Sri_Lanka ......................T................. [120]

pallidus-Sri_Lanka-1 ......................T................. [120]

annularis-FJ526544.1-Laos CATGGCGCAAGCCTTCGTCGGACCCCTCCATCCCGGGGTG [160]

annularis-FJ526541.1-Myanmar-1 ........................................ [160]

annularis-FJ526537.1-Myanmar-2 ........................................ [160]

annularis-FJ526542.1-Philippines ........................................ [160]

annularis-FJ526545.1-Thailand ........................................ [160]

annularis-FJ526543.1-Viet_Nam ........................................ [160]

annularis-FJ526536.1-Sri_Lanka ........................................ [160]

annularis-Sri_Lanka-3 ........................................ [160]

annularis-Sri_Lanka-1 ........................................ [160]

annularis-Sri_Lanka-2 ........................................ [160]

annularis_spA-DQ351855.1-India ........................................ [160]

annularis_spB-DQ351856.1-India ........................................ [160]

pallidus-FJ526535.1-Sri_Lanka .....................G.................. [160]

pallidus-Sri_Lanka-1 .....................G.................. [160]

annularis-FJ526544.1-Laos TCCCGCTACGGGCTGTGTTCGCGTCTCACGATGCGTTCGC [200]

annularis-FJ526541.1-Myanmar-1 ........................................ [200]

annularis-FJ526537.1-Myanmar-2 ........................................ [200]

annularis-FJ526542.1-Philippines ........................................ [200]

annularis-FJ526545.1-Thailand ........................................ [200]

annularis-FJ526543.1-Viet_Nam ........................................ [200]

annularis-FJ526536.1-Sri_Lanka ........................................ [200]

annularis-Sri_Lanka-3 ........................................ [200]

annularis-Sri_Lanka-1 ........................................ [200]

annularis-Sri_Lanka-2 ........................................ [200]

annularis_spA-DQ351855.1-India ........................................ [200]

annularis_spB-DQ351856.1-India ......................T................. [200]

pallidus-FJ526535.1-Sri_Lanka ....AT.T............A.TCY.....GG.T...... [200]

pallidus-Sri_Lanka-1 ....AT.T............A.TCT.....GG.T...... [200]

annularis-FJ526544.1-Laos TGTCCCGTGTGGGCATCCCTCGAGTGCGTAGGATGCGACC [240]

annularis-FJ526541.1-Myanmar-1 ........................................ [240]

annularis-FJ526537.1-Myanmar-2 ........................................ [240]

annularis-FJ526542.1-Philippines ........................................ [240]

annularis-FJ526545.1-Thailand ........................................ [240]

annularis-FJ526543.1-Viet_Nam ........................................ [240]

annularis-FJ526536.1-Sri_Lanka ........................................ [240]

annularis-Sri_Lanka-3 ........................................ [240]

annularis-Sri_Lanka-1 ........................................ [240]

annularis-Sri_Lanka-2 ........................................ [240]

annularis_spA-DQ351855.1-India ........................................ [240]

annularis_spB-DQ351856.1-India ........................................ [240]

pallidus-FJ526535.1-Sri_Lanka .-.....G................................ [240]

pallidus-Sri_Lanka-1 .-.....G................................ [240]

annularis-FJ526544.1-Laos CGAAAGATGGTGAACTATGCCTGATCAGGCCGAAGTCAGG [280]

annularis-FJ526541.1-Myanmar-1 ........................................ [280]

annularis-FJ526537.1-Myanmar-2 ........................................ [280]

annularis-FJ526542.1-Philippines ........................................ [280]

annularis-FJ526545.1-Thailand ........................................ [280]

annularis-FJ526543.1-Viet_Nam ........................................ [280]

annularis-FJ526536.1-Sri_Lanka ........................................ [280]

annularis-Sri_Lanka-3 ........................................ [280]

annularis-Sri_Lanka-1 ........................................ [280]

annularis-Sri_Lanka-2 ........................................ [280]

annularis_spA-DQ351855.1-India ........................................ [280]

annularis_spB-DQ351856.1-India ........................................ [280]

pallidus-FJ526535.1-Sri_Lanka ........................................ [280]

pallidus-Sri_Lanka-1 ........................................ [280]

annularis-FJ526544.1-Laos GGAAACCCTGATGGAGGGCCGAAGCAATTCTGACGTGCAA [320]

annularis-FJ526541.1-Myanmar-1 ........................................ [320]

annularis-FJ526537.1-Myanmar-2 ........................................ [320]

annularis-FJ526542.1-Philippines ........................................ [320]

annularis-FJ526545.1-Thailand ........................................ [320]

annularis-FJ526543.1-Viet_Nam ........................................ [320]

annularis-FJ526536.1-Sri_Lanka ........................................ [320]

annularis-Sri_Lanka-3 ........................................ [320]

annularis-Sri_Lanka-1 ........................................ [320]

annularis-Sri_Lanka-2 ........................................ [320]

annularis_spA-DQ351855.1-India ........................................ [320]

annularis_spB-DQ351856.1-India ........................................ [320]

pallidus-FJ526535.1-Sri_Lanka ........................................ [320]

pallidus-Sri_Lanka-1 ........................................ [320]

annularis-FJ526544.1-Laos ATCGATTGTCAGAGTTGGGCATAGGGGCGAAAGACCAATC [360]

annularis-FJ526541.1-Myanmar-1 ........................................ [360]

annularis-FJ526537.1-Myanmar-2 ........................................ [360]

annularis-FJ526542.1-Philippines ........................................ [360]

annularis-FJ526545.1-Thailand ........................................ [360]

annularis-FJ526543.1-Viet_Nam ........................................ [360]

annularis-FJ526536.1-Sri_Lanka ........................................ [360]

annularis-Sri_Lanka-3 ........................................ [360]

annularis-Sri_Lanka-1 ........................................ [360]

annularis-Sri_Lanka-2 ........................................ [360]

annularis_spA-DQ351855.1-India ........................................ [360]

annularis_spB-DQ351856.1-India ........................................ [360]

pallidus-FJ526535.1-Sri_Lanka ........................................ [360]

pallidus-Sri_Lanka-1 ........................................ [360]

annularis-FJ526544.1-Laos ----------------------------- [389]

annularis-FJ526541.1-Myanmar-1 ----------------------------- [389]

annularis-FJ526537.1-Myanmar-2 ----------------------------- [389]

annularis-FJ526542.1-Philippines ----------------------------- [389]

annularis-FJ526545.1-Thailand ----------------------------- [389]

annularis-FJ526543.1-Viet_Nam ----------------------------- [389]

annularis-FJ526536.1-Sri_Lanka ----------------------------- [389]

annularis-Sri_Lanka-3 GAACCATCTAGTAGCTGGTTCCTTCCGAA [389]

annularis-Sri_Lanka-1 GAACCATCTAGTAGCTGGTTCCTTCCGAA [389]

annularis-Sri_Lanka-2 GAACCATCTAGTAGCTGGTTCCTTCCGA- [389]

annularis_spA-DQ351855.1-India GAACCATCTAGTAGCTGGTTCCTTCCGA- [389]

annularis_spB-DQ351856.1-India GAACCATCTAGTAGCTGGTTCCTTCCGA- [389]

pallidus-FJ526535.1-Sri_Lanka ----------------------------- [389]

pallidus-Sri_Lanka-1 GAACCATCTAGTAGCTGGTTCCTTCCGAG [389]
