# Supplementary material for: Molecular evidence for the presence of malaria vector species a of the Anopheles annularis complex in Sri Lanka
Source: Parasit Vectors. 2011 Dec 22;4:239. doi: 10.1186/1756-3305-4-239 (PMC3293028; doi:10.1186/1756-3305-4-239)
Supplement: Additional file 2 — ITS-2 sequences used for phylogenetic analysis. ITS-2 sequences used for phylogenetic analysis presented in Figure 2. Sri Lanka - 1, 2 and 3 refers to individual specimens collected in this study. Other sequences of An. pallidus and An. annularis from different countries were obtained from GenBank. [file 1756-3305-4-239-S2.DOC]

**Supplementary File 2: ITS-2 sequences used for phylogenetic analysis**

annularis-EU919720.1-China ---------------------------------GAACTGC [ 40]

annularis-FJ526609.1-Myanmar ATGAAGACCGCAGCTAAATGCGCGTCAGAATGT....... [ 40]

annularis-FJ526613.1-Philippines ATGAAGACCGCAGCTAAATGCGCGTCAGAATGT....... [ 40]

annularis-FJ526607.1-Sri_Lanka ATGAAGACCGCAGCTAAATGCGCGTCAGAATGT....... [ 40]

annularis-FJ526615.1-Thailand ATGAAGACCGCAGCTAAATGCGCGTCAGAATGT....... [ 40]

annularis-GU062187.1-Timor -------------------------------------... [ 40]

annularis_spA-DQ351853.1-India ---------------------------------------- [ 40]

annularis_spB-DQ351854.1-India ---------------------------------------- [ 40]

annularis-Sri_Lanka-1 ---------------------------------------- [ 40]

annularis-Sri_Lanka-2 ---------------------------------------- [ 40]

annularis-Sri_Lanka-3 ---------------------------------------- [ 40]

pallidus-FJ526606.1-Sri_Lanka ATGAAGACCGCAGCTAAATGCGCGTCAGAATGT....... [ 40]

pallidus-Sri_Lanka-1 ---------------------------------------- [ 40]

annularis-EU919720.1-China AGGACACATGAACATTGATAAGTTGAACGCATATTGCGCA [ 80]

annularis-FJ526609.1-Myanmar ........................................ [ 80]

annularis-FJ526613.1-Philippines ........................................ [ 80]

annularis-FJ526607.1-Sri_Lanka ........................................ [ 80]

annularis-FJ526615.1-Thailand ........................................ [ 80]

annularis-GU062187.1-Timor ........................................ [ 80]

annularis_spA-DQ351853.1-India ----------.............................. [ 80]

annularis_spB-DQ351854.1-India ----------.............................. [ 80]

annularis-Sri_Lanka-1 -----------------------------------..... [ 80]

annularis-Sri_Lanka-2 --------------------...--G.............. [ 80]

annularis-Sri_Lanka-3 -----------------------------------..... [ 80]

pallidus-FJ526606.1-Sri_Lanka ..............CC..C.C................... [ 80]

pallidus-Sri_Lanka-1 ---------------------------------------- [ 80]

annularis-EU919720.1-China TCGGACGTCTCAACCCGACCGATGCACACATCCTTGAGTG [120]

annularis-FJ526609.1-Myanmar ........................................ [120]

annularis-FJ526613.1-Philippines ........................................ [120]

annularis-FJ526607.1-Sri_Lanka ........................................ [120]

annularis-FJ526615.1-Thailand ........................................ [120]

annularis-GU062187.1-Timor ........................................ [120]

annularis_spA-DQ351853.1-India ........................................ [120]

annularis_spB-DQ351854.1-India ........................................ [120]

annularis-Sri_Lanka-1 ............-........................... [120]

annularis-Sri_Lanka-2 ........................................ [120]

annularis-Sri_Lanka-3 ............-........................... [120]

pallidus-FJ526606.1-Sri_Lanka ........T......................T........ [120]

pallidus-Sri_Lanka-1 ---------------------------------------- [120]

annularis-EU919720.1-China CCTACCAAGTTATCTATACGCATTTACCAAACTGACAGCC [160]

annularis-FJ526609.1-Myanmar ........................................ [160]

annularis-FJ526613.1-Philippines ........................................ [160]

annularis-FJ526607.1-Sri_Lanka ........................................ [160]

annularis-FJ526615.1-Thailand ........................................ [160]

annularis-GU062187.1-Timor ........................................ [160]

annularis_spA-DQ351853.1-India ........................................ [160]

annularis_spB-DQ351854.1-India ........................................ [160]

annularis-Sri_Lanka-1 ........................................ [160]

annularis-Sri_Lanka-2 ........................................ [160]

annularis-Sri_Lanka-3 ........................................ [160]

pallidus-FJ526606.1-Sri_Lanka ...................A.TC.....T........A.- [160]

pallidus-Sri_Lanka-1 -------------......A.TC.....T........A.- [160]

annularis-EU919720.1-China TATCTTCGGGATAGAT--GTCGCATCATGGCGTGCTCGGA [200]

annularis-FJ526609.1-Myanmar .....C..........--...................... [200]

annularis-FJ526613.1-Philippines .....C..........--...................... [200]

annularis-FJ526607.1-Sri_Lanka .....C..........--...................... [200]

annularis-FJ526615.1-Thailand ................--...................... [200]

annularis-GU062187.1-Timor ................--...................... [200]

annularis_spA-DQ351853.1-India .....C..........--...................... [200]

annularis_spB-DQ351854.1-India ................--...................... [200]

annularis-Sri_Lanka-1 .....C..........--...................... [200]

annularis-Sri_Lanka-2 .....C..........--...................... [200]

annularis-Sri_Lanka-3 .....C..........--...................... [200]

pallidus-FJ526606.1-Sri_Lanka .....GG.A....AGCCA.......AT............. [200]

pallidus-Sri_Lanka-1 .....GG.A....AGCCA.......AT............. [200]

annularis-EU919720.1-China CCCGCACTGGACGGGACCTTGGGCGCTGAAAGTGTGAAAG [240]

annularis-FJ526609.1-Myanmar ........................................ [240]

annularis-FJ526613.1-Philippines ........................................ [240]

annularis-FJ526607.1-Sri_Lanka ........................................ [240]

annularis-FJ526615.1-Thailand ...................................A.... [240]

annularis-GU062187.1-Timor ...................................A.... [240]

annularis_spA-DQ351853.1-India ........................................ [240]

annularis_spB-DQ351854.1-India ........................................ [240]

annularis-Sri_Lanka-1 ........................................ [240]

annularis-Sri_Lanka-2 ........................................ [240]

annularis-Sri_Lanka-3 ........................................ [240]

pallidus-FJ526606.1-Sri_Lanka ......T........G..................A..G.. [240]

pallidus-Sri_Lanka-1 ......T........G..................A..G.. [240]

annularis-EU919720.1-China CTAATACAGATGACTTTGTATGGTAAGG------------ [280]

annularis-FJ526609.1-Myanmar ............................------------ [280]

annularis-FJ526613.1-Philippines ............................------------ [280]

annularis-FJ526607.1-Sri_Lanka ............................------------ [280]

annularis-FJ526615.1-Thailand ............................------------ [280]

annularis-GU062187.1-Timor ............................------------ [280]

annularis_spA-DQ351853.1-India ............................------------ [280]

annularis_spB-DQ351854.1-India ............................------------ [280]

annularis-Sri_Lanka-1 ............................------------ [280]

annularis-Sri_Lanka-2 ............................------------ [280]

annularis-Sri_Lanka-3 ............................------------ [280]

pallidus-FJ526606.1-Sri_Lanka ......T...A...AA.....C..G...TACAAGGTAGCA [280]

pallidus-Sri_Lanka-1 ......T...A...AA.....C..G...TACAAGGTAGCA [280]

annularis-EU919720.1-China -GTAAGCGATGAACAGACACCGCGACAAGTCGCA-TGGTT [320]

annularis-FJ526609.1-Myanmar -.................................-..... [320]

annularis-FJ526613.1-Philippines -.................................-..... [320]

annularis-FJ526607.1-Sri_Lanka -.................................-..... [320]

annularis-FJ526615.1-Thailand -.................................-..... [320]

annularis-GU062187.1-Timor -..........G......................-..... [320]

annularis_spA-DQ351853.1-India -.................................-..... [320]

annularis_spB-DQ351854.1-India -..G..............-T..............-..... [320]

annularis-Sri_Lanka-1 -.................................-..... [320]

annularis-Sri_Lanka-2 -.......-------------------------------- [320]

annularis-Sri_Lanka-3 -.................................-..... [320]

pallidus-FJ526606.1-Sri_Lanka A..G..A.....GA.....-..............A..... [320]

pallidus-Sri_Lanka-1 A..G..A.....GA.....-..............A..... [320]

annularis-EU919720.1-China CGACCTCA-GTATCAACCTGGGATGAAACCCCCGCAGCCT [360]

annularis-FJ526609.1-Myanmar ........-.........C.....C............... [360]

annularis-FJ526613.1-Philippines ........-.........C.....C............... [360]

annularis-FJ526607.1-Sri_Lanka ........-.........C.....C............... [360]

annularis-FJ526615.1-Thailand ........-............................... [360]

annularis-GU062187.1-Timor ........-.........C..................... [360]

annularis_spA-DQ351853.1-India ........-.........C.....C............... [360]

annularis_spB-DQ351854.1-India ........-.........C..................... [360]

annularis-Sri_Lanka-1 ........-.........C.....C............... [360]

annularis-Sri_Lanka-2 ---------------------------------------- [360]

annularis-Sri_Lanka-3 ........-.........C.....C............... [360]

pallidus-FJ526606.1-Sri_Lanka ........A........TC..................... [360]

pallidus-Sri_Lanka-1 ........A........TC..................... [360]

annularis-EU919720.1-China ATCA-CGGGCGCTAGCAAAGGGGTCCTGGGTGG-CTCTGG [400]

annularis-FJ526609.1-Myanmar ....-.........................G..G...... [400]

annularis-FJ526613.1-Philippines ....-.........................G..G...... [400]

annularis-FJ526607.1-Sri_Lanka ....-.........................G..G...... [400]

annularis-FJ526615.1-Thailand ....-............................-...... [400]

annularis-GU062187.1-Timor ....-......................A.....-...... [400]

annularis_spA-DQ351853.1-India ....-.........................G..G...... [400]

annularis_spB-DQ351854.1-India ....-......................A.....-...... [400]

annularis-Sri_Lanka-1 ....-.........................G..G...... [400]

annularis-Sri_Lanka-2 ---------------------------------------- [400]

annularis-Sri_Lanka-3 ....-.........................G..G...... [400]

pallidus-FJ526606.1-Sri_Lanka ....T.A..........................-...G.. [400]

pallidus-Sri_Lanka-1 ....T.A..........................-...G.. [400]

annularis-EU919720.1-China TCGTGTAACACTTGCGGCCCAACGGGTCTGTCTTCATCGG [440]

annularis-FJ526609.1-Myanmar .............................A.......... [440]

annularis-FJ526613.1-Philippines .............................A.......... [440]

annularis-FJ526607.1-Sri_Lanka ........................................ [440]

annularis-FJ526615.1-Thailand ........................................ [440]

annularis-GU062187.1-Timor ........................................ [440]

annularis_spA-DQ351853.1-India ........................................ [440]

annularis_spB-DQ351854.1-India ........................................ [440]

annularis-Sri_Lanka-1 ........................................ [440]

annularis-Sri_Lanka-2 ---------------------------------------- [440]

annularis-Sri_Lanka-3 ........................................ [440]

pallidus-FJ526606.1-Sri_Lanka ...........................----...T....T [440]

pallidus-Sri_Lanka-1 ...........................----...T....T [440]

annularis-EU919720.1-China CTTGCA---------------------------------- [480]

annularis-FJ526609.1-Myanmar ......AAG--GCAAACACGGGAATTGGAAAATTCTCTAT [480]

annularis-FJ526613.1-Philippines ......AAG--GCAAACACGGGAATTGGAAAATTCTCTAT [480]

annularis-FJ526607.1-Sri_Lanka ......AAG--GCAAACACGGGAATTGGAAAATTCTCTAT [480]

annularis-FJ526615.1-Thailand ......AAG--GCAAACACGGGAATTGGAAAATTCTCTAT [480]

annularis-GU062187.1-Timor ......AAC--GCAAACACGGGAATTGGAAAATTCTCTAT [480]

annularis_spA-DQ351853.1-India ......AAG--GCAAACACGGGAATTGGAAAATTCTCTAT [480]

annularis_spB-DQ351854.1-India ......AACACGCAAACACGGGAATTGGAAAATTCTCTAT [480]

annularis-Sri_Lanka-1 ......AAG--GCAAACACGGGAATTGGAAAATTCTCTAT [480]

annularis-Sri_Lanka-2 ---------------------------------------- [480]

annularis-Sri_Lanka-3 ......AAG--GCAAACACGGGAATTGGAAAATTCTCTAT [480]

pallidus-FJ526606.1-Sri_Lanka ..CA.TTAA---CATAAACGGGAATTAGC--TTCTCTCAT [480]

pallidus-Sri_Lanka-1 ..CA.TTAA---CATAAACGGGAATTAGC--TTCTCTCAT [480]

annularis-EU919720.1-China ---------------------------------------- [520]

annularis-FJ526609.1-Myanmar GTAGGCCTCAAGTGATGTGT-------------------- [520]

annularis-FJ526613.1-Philippines GTAGGCCTCAAGTGATGTGT-------------------- [520]

annularis-FJ526607.1-Sri_Lanka GTAGGCCTCAAGTGATGTGT-------------------- [520]

annularis-FJ526615.1-Thailand GTAGGCCTCAAGTGATGTGT-------------------- [520]

annularis-GU062187.1-Timor GTAGGCCTCAAGTGATGTGTGACAACCCCCAGAATTTAAG [520]

annularis_spA-DQ351853.1-India GTAGGCCTCAAGTGATGTGTGA------------------ [520]

annularis_spB-DQ351854.1-India GTAGGCCTCAAGTGATGTGTGA------------------ [520]

annularis-Sri_Lanka-1 GTAGGCCTCAAGTGATGTGTGACAACCCCCTGAATTTAAG [520]

annularis-Sri_Lanka-2 ---------------------------------------- [520]

annularis-Sri_Lanka-3 GTAGGCCTCAAGTGATGTGTGACAACCCCCTGAATTTAAG [520]

pallidus-FJ526606.1-Sri_Lanka GTAGGCCTCAAGTGATGTGT-------------------- [520]

pallidus-Sri_Lanka-1 GTAGGCCTCAAGTGATGTGGGACAACCCCCTGAATATAAG [520]

annularis-EU919720.1-China ---------------------------------------- [560]

annularis-FJ526609.1-Myanmar ---------------------------------------- [560]

annularis-FJ526613.1-Philippines ---------------------------------------- [560]

annularis-FJ526607.1-Sri_Lanka ---------------------------------------- [560]

annularis-FJ526615.1-Thailand ---------------------------------------- [560]

annularis-GU062187.1-Timor CATATTAATAAGGGGAGGAAGAGAAACCAACCGGGATTCC [560]

annularis_spA-DQ351853.1-India ---------------------------------------- [560]

annularis_spB-DQ351854.1-India ---------------------------------------- [560]

annularis-Sri_Lanka-1 CATAACCC-------------------------------- [560]

annularis-Sri_Lanka-2 ---------------------------------------- [560]

annularis-Sri_Lanka-3 CATAACCC-------------------------------- [560]

pallidus-FJ526606.1-Sri_Lanka ---------------------------------------- [560]

pallidus-Sri_Lanka-1 CATAAA---------------------------------- [560]

annularis-EU919720.1-China ---------------- [576]

annularis-FJ526609.1-Myanmar ---------------- [576]

annularis-FJ526613.1-Philippines ---------------- [576]

annularis-FJ526607.1-Sri_Lanka ---------------- [576]

annularis-FJ526615.1-Thailand ---------------- [576]

annularis-GU062187.1-Timor CTGAGTAGCTGCGGCG [576]

annularis_spA-DQ351853.1-India ---------------- [576]

annularis_spB-DQ351854.1-India ---------------- [576]

annularis-Sri_Lanka-1 ---------------- [576]

annularis-Sri_Lanka-2 ---------------- [576]

annularis-Sri_Lanka-3 ---------------- [576]

pallidus-FJ526606.1-Sri_Lanka ---------------- [576]

pallidus-Sri_Lanka-1 ---------------- [576]
